# Effect of Back-Tempering on the Wear and Corrosion Properties of Multiple-Pass Friction Stir Processed High-Speed Steel

**DOI:** 10.3390/ma18174125

**Published:** 2025-09-02

**Authors:** Quan Liu, Shiye Li, Guochong Rao, Xiaomi Chen, Kun Liu, Min Zhou, Dawei Guo, Valentino A. M. Cristino, Kin-Ho Lo, Lap-Mou Tam, Chi-Tat Kwok

**Affiliations:** 1 Department of Electromechanical Engineering, Faculty of Science and Technology, University of Macau, Macao, China; yc47948@um.edu.mo (Q.L.); mc45336@um.edu.mo (S.L.); mc45240@um.edu.mo (G.R.); yc17474@um.edu.mo (X.C.); yc17923@um.edu.mo (K.L.); vcristino@um.edu.mo (V.A.M.C.); fstkhl@um.edu.mo (K.-H.L.); fstlmt@um.edu.mo (L.-M.T.); 2 IDQ Science and Technology (Hengqin, Guangdong) Co., Ltd., Hengqin 519031, China; dwguo@idq.org.mo; 3 Institute for the Development and Quality, Macao, China; 4 Institute of Applied Physics and Materials Engineering, University of Macau, Macao, China

**Keywords:** friction stir processing, back-tempering, high-speed steel, wear, corrosion

## Abstract

In this study, a scalable surface modification strategy for M2 high-speed steel was applied using multiple-pass friction stir processing (FSP) with overlapping ratios of 25%, 50%, and 75%. A comprehensive investigation of the microstructure, surface hardness, wear, and corrosion resistance was conducted to elucidate the properties of FSPed M2 as a function of the overlapping ratio. In the single-pass FSPed M2, the major phase was martensite and the minor phases included retained austenite where refined carbides (M_6_C, M_23_C_6_, and MC) were detected. However, back-tempering occurred near the overlapped zone (OZ) between consecutive tracks for the multiple-pass FSPed M2. The martensite formed in the first pass was turned into tempered martensite by the thermal cycle from the subsequent pass. This back-tempering resulted in a localized decline in hardness from 900 to 650 HV_0.2_. Further wear tests revealed that the wear rates of the tempered zone (TZ) of the multiple-pass FSPed M2 (FSP25%: 1.40 × 10^−5^ mm^3^/N·m, FSP50%: 1.20 × 10^−5^ mm^3^/N·m and FSP75%: 1.00 × 10^−5^ mm^3^/N·m) are all higher than that of SZ of the single-pass FSPed M2 (0.75 × 10^−5^ mm^3^/N·m), indicating lower wear resistance of the TZ. Moreover, increased carbide content in the TZ led to the depletion of passivating elements near proximity of the tempered martensite, acting as the active sites for selective corrosion attack. The corrosion potential (E_corr_) and corrosion current density (I_corr_) increased significantly, with values of −397.6 ± 5.6 mV and 9.5 ± 0.8 μA·cm^−2^ for FSP25%, −424.4 ± 6.0 mV and 14.7 ± 1.7 μA·cm^−2^ for FSP50%, and −440.9 ± 2.8 mV and 17.1 ± 1.9 μA·cm^−2^ for FSP75%.

## 1. Introduction

M2 is a type of high-speed steel (HSS), widely used in making various high-speed cutting tools and molds due to its excellent thermal stability, strength, impact toughness, high-temperature hardness, and wear resistance [1,2]. It can maintain the sharpness and durability of the cutting edge in high-speed and heavy-duty cutting applications. Compared with ordinary tool steels, M2 contains a high carbon content (0.78–1.05 wt%) and alloying elements, including molybdenum, tungsten, vanadium, and chromium, which contribute to the formation of metal carbides. It is widely used to make cutting tools, gears, bearings, molds, crankshafts, springs, aircraft engine parts, missile components, and rocket engine parts [3].

Friction stir welding (FSW), an innovative solid-state joining technique, was first developed in 1991 by the Welding Institute (TWI) in the United Kingdom [4]. Friction stir processing (FSP) is an offshoot of FSW and a solid-state processing technology, and was pioneered by Mishra et al. [4]. It has emerged as a transformative surface modification technique. By adjusting processing parameters, it allows precise control over the microstructure of material, thereby enhancing surface properties. FSP employs a non-consumable rotating tool comprising a specially designed pin and shoulder that penetrates the materials, which leads to frictional heating and severe plastic deformation, thereby refining the grains and homogenizing the material’s microstructure [5]. Pan et al. performed FSP on martensitic stainless steels such as AISI 420 [6] and 440C [7] by the tool without a pin. Under the optimal processing parameters at 2000 rpm and 150 mm/min, the FSPed AISI 420 showed high hardness (697 HV_1_) with concurrently enhanced corrosion resistance, while FSPed AISI 440C showed higher hardness up to 779 HV_1_ and excellent pitting corrosion resistance. Sorensen et al. [8] used FSP to fabricate D2 steel blade blanks and manufacture the blade tools. The edge life of FSPed D2 blade tools was found to be increased by 10 times compared to the traditional thermo-mechanical processed and heat-treated ones, primarily due to the significantly refined grain size (500 nm). In addition, the stir zone exhibited a higher concentration of chromium and carbon in the martensite, leading to a hardness exceeding 1000 HV_0.5_. This elevated hardness facilitates the creation of an exceptionally sharp edge. Ma et al. [9] demonstrated that ultrasonic-assisted FSP of cast A356 aluminum alloy significantly enhanced grain refinement and uniformity, leading to improved hardness, tensile strength, and ductility at fracture. In addition to tool steels, FSP has successfully modified the surface and optimizing the performance of various materials, including aluminum alloys [10,11,12], magnesium alloys [13,14], copper and its alloys [15,16], stainless steels [6,7,17,18], and polymers [19].

Single-pass FSP can only process a limited area of the components due to the limited dimensions of the pin and shoulder of the mechtrode. Therefore, a larger processed area can be achieved through multiple-pass FSP by overlapping the successive single tracks. Nonetheless, only a few researchers have conducted studies on multiple-pass FSP of steels [20,21], high entropy alloy (HEA) [22] and stainless steels [17,18,23]. Aldajah et al. [21] reported that multiple-pass FSP can improve the wear resistance of high-carbon steel, thereby alleviating severe plastic deformation, suppressing material degradation, and decreasing wear loss. Pan et al. [23] investigated the effects of multiple-pass FSP on the microstructure, corrosion resistance, and hardness of AISI 420 martensitic stainless steel. They found that microstructural changes by tempering near the overlapping zone resulted in a reduction in localized hardness and corrosion resistance in this region. Raja and their colleagues [20] investigate the impact of multiple-pass FSP on IS2062 steel, revealing significant improvements in micro-hardness and grain refinement (average grain size of 22 μm) to 175 VH0.2 after the second-pass compared to the unprocessed material (130 VH_0.2_, 57 mm). Multiple-pass FSP of Fe_49.5_Mn_30_Co_10_Cr_10_C_0.5_ HEA [22] revealed progressive grain refinement and hexagonal close-packed (HCP) phase evolution with each pass, significantly influencing its mechanical properties, with one-pass FSP yielding the highest ultimate tensile strength (UTS) and work hardening rate due to its fine grains and high metastable face-centered cubic (FCC) phase fraction. In contrast, the double-pass FSP was characterized by a higher content of HCP phase with finer grains and superior yield strength compared to one-pass FSPed specimen.

In the literature, no research on the effect of multiple-pass FSP on HSSs is reported. In this study, the microstructure, hardness, corrosion and wear behaviors of AISI M2 HSS processed by multiple-pass FSP were investigated. Moreover, different degrees of overlapping (25%, 50%, and 75%) of the consecutive single-pass were conducted. FSP enabled the formation of a martensitic matrix reinforced by uniformly distributed fine carbides, thereby enhancing the mechanical properties of M2 HSS. The innovation of this study lies in the use of a pin-type mechtrode to achieve a wide coverage and deep penetration of the FSPed M2 while investigating the effects of multiple-pass FSP with different overlapping ratios on the hardness, wear and corrosion behavior, and comparison to annealed, conventionally hardened, and single-pass FSPed M2. Notably, the transition zone may lead to localized degradation and plays a critical role in the changes in hardness, wear, and corrosion resistance, making it essential to investigate.

## 2. Materials and Methods

### 2.1. Materials

The as-received AISI M2 HSS (AR M2) (Shanghai Huaxiao Metal Materials, Shanghai, China) was in an annealed state with a dimension of 100 × 50 × 5 mm^3^, and its chemical compositions of AISI M2 HSS were analyzed using the spark source atomic emission spectroscopy analysis (SPECTRO LAM M10, SPECTRO Analytical Instruments GmbH, Kleve, Germany) method listed in Table 1. For the mechanical and thermal properties of the AISI M2 [24], it has an elastic modulus of 210 GPa and a Poisson’s ratio of 0.3. The compressive yield strength reaches 3250 MPa. Its thermal conductivity increases with temperature, from 21.3 W/m·K at 95 °C to 23.5 W/m·K at 200 °C, 25.6 W/m·K at 400 °C, and 27 W/m·K at 540 °C. The specific heat capacity is 55 J/kg·K, and the density is 8150 kg/m^3^. The coefficient of thermal expansion in the range of 20–540 °C is 11.9 × 10^−6^/K. It was austenized at 1160 °C for 30 min, followed by rapid cooling in oil. Then, it was followed by tempering three times at 560 °C, with each session lasting 2 h and finally quenched in oil. The conventionally quenched and tempered M2 is designated as QT M2.

### 2.2. Friction Stir Processing

**Figure 1:**
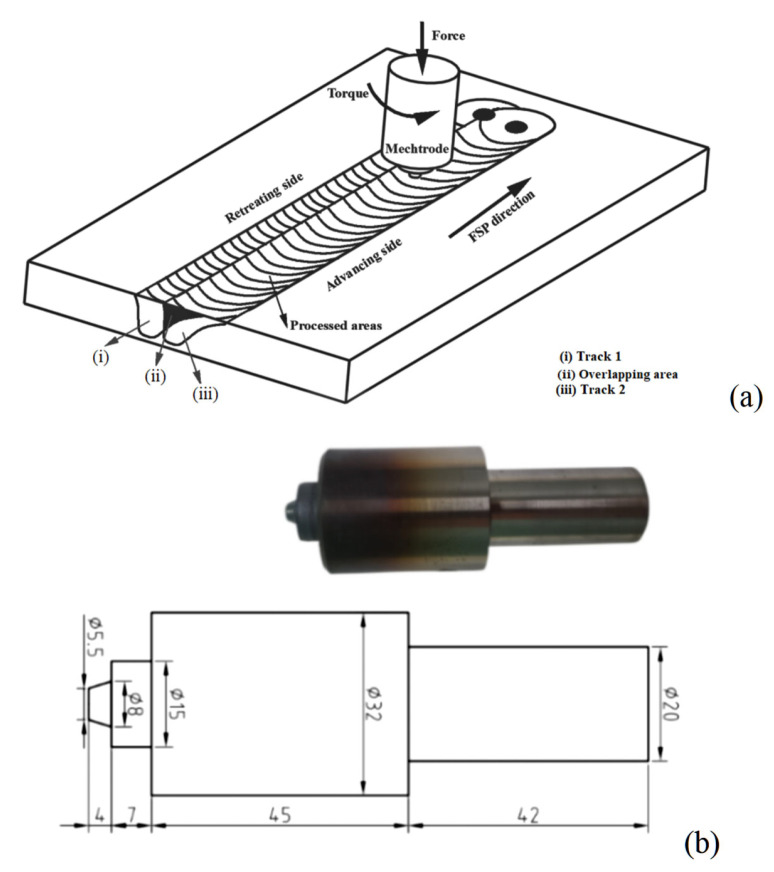
(**a**) Schematic representation of multiple-pass FSP, and (**b**) photograph and dimensions of mechtrode used for FSP (Φ represents the diameter).

In this study, FSP of AISI M2 HSS was conducted using commercial FSW equipment (FSW-TS-M16, FSW Center, Beijing, China). Figure 1 depicts the schematic representation of multiple-pass FSP with a mechtrode containing a 15-mm diameter shoulder, and a 4-mm long threaded and tapered pin with a root diameter of 5.5 mm and a tip diameter of 8 mm. The FSP tool was fabricated from a tungsten–rhenium (W-Re) alloy. During FSP, the mechtrode was titled at an angle of 3°, which was beneficial to fully forge the material. The FSP parameters were optimized as shown in Table 2. Notably, a rotation speed of 600 rpm and a traverse speed of 100 mm/min were selected based on observations of a microscopically defect-free stir zone and low surface roughness.

**Figure 2:**
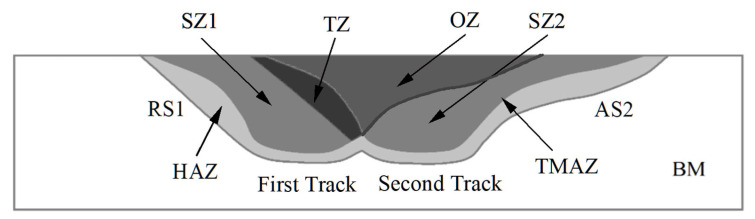
Schematic diagram of the cross-section of multiple-pass FSPed M2. OZ: overlapped zone of first track and second track; TZ: tempered zone; RS1: retreating side of first track; SZ1: stir zone of first track; AS2: advancing side of second track; SZ2: stir zone of second track; TMAZ: thermal mechanically affected zone; HAZ: heat affected zone; BM: base material.

To improve the surface properties of M2 HSS through a large area, multiple-pass FSP was implemented and studied. Figure 2 shows the schematic diagram of the cross-section of multiple-pass FSP. After the first-pass FSP, the second-pass FSP was performed using the same processing parameters with different overlapping ratios (ORs). The OR is defined as follows [15]:

OR = 1 − (x/d)(1)

where x is the distance between the centers of each pass, and d represents the diameter of the shoulder. The ORs attempted in this study are 25%, 50%, and 75%.

### 2.3. Metallographic and Microstructural Studies

The cross-sections and surfaces of the single-pass and multiple-pass FSPed M2, AR M2, and QT M2 were sequentially ground using different CAMI SiC papers with grit sizes of #400, #800, #1000, #2500, and #5000. Subsequent polishing was performed with 1 μm diamond paste, cleaned with ethanol, and etched with acidified ferric chloride solution composed of H_2_O of 100 mL, HCl of 25 mL, and FeCl_3_ of 25 g. Microstructural and compositional analyses were performed using an optical microscope and a scanning electron microscope (SEM, S-3400N, Hitachi, Tokyo, Japan) equipped with energy disperse spectroscopy (EDS, EX-250, Horiba, Tokyo, Japan). Phase identification was carried out via X-ray diffraction (XRD, MiniFlex 600, Rigaku, Neu-Isenburg, Germany) operated at 40 kV and 15 mA, scanned at a rate of 3°/min with a diffraction angle (2θ) from 20° to 140°.

### 2.4. Hardness Testing

Hardness measurements for different samples were conducted utilizing a Vickers hardness tester (Qness 60A EVO, ATM Qness, Mammelzen, Germany). The parameters for hardness tests are chosen at a 200-g load and a 10-s duration, conforming to ASTM E384-17 [25]. A minimum of three tests were carried out longitudinally along the center lines of each pass in multiple-pass FSPed areas (depth direction) and transversely from the retreating side (RS) to advancing side (AS) at 1 mm below the top surface.

### 2.5. Wear Testing

A wear test was performed using a UMT TriboLab tribometer (Bruker TL-18-12-175, Berlin, Germany) to determine the coefficient of friction (COF) and wear resistance of AR M2, QT M2, single-pass, and multiple-pass FSPed M2 at 25 °C under dry sliding conditions conforming to ASTM G133-05 [26]. The test was conducted using a 9.5 mm diameter alumina ball (the counter-body). The tests employed a 200 N load, a 10 mm stroke length, and a 5 Hz oscillation frequency. The total sliding duration was set to 1000 s for both FSPed specimens and 200 s for the AR M2 and QT M2. Wear depth and wear volume were measured using a 3D optical profilometer (Contour GT-X3, Bruker, Berlin, Germany), and worn surfaces were analyzed using the SEM. Wear rate was calculated according to [22] as wear volume (mm^3^) divided by the product of applied load (N) and total sliding distance (m).

### 2.6. Corrosion Testing

All specimens for corrosion tests (15 × 15 × 5 mm^3^) were mounted in the cold-curing epoxy resin, sequentially polished to 1000-grit using SiC paper, ultrasonically cleaned in ethanol, and finally dried. After sealing the gap between the epoxy resin and the specimens, an area of 1 cm^2^ was exposed to 3.5 wt% NaCl solution at 25 °C, opened in air. A three-electrode cell (VersaSTAT 3, Princeton, NJ, USA) was employed for the electrochemical measurements with the specimens as the working electrode, a saturated calomel electrode (SCE) as the reference electrode, and graphite rods as the counter electrodes. After being immersed in the NaCl solution for 30 min, the open circuit potential (OCP) of the specimen was stabilized, then the potentiodynamic polarization (PD) and electrochemical impedance spectroscopy (EIS) measurement were performed separately. For the PD tests, the potential was scanned from −0.25 V_SCE_ below the OCP to +0.25 V_SCE_ with a scan rate of 0.5 mV_SCE_/s. The corrosion potential (E_corr_) and corrosion current (I_corr_) were determined via Tafel extrapolation using the software named Versa 2.4.2 (Princeton, NJ, USA). EIS tests were measured at OCP with a sinusoidal potential perturbation of 10 mV and a frequency from 100 kHz to 0.01 Hz, with data fitted using ZView2 software (Princeton, NJ, USA). All electrochemical tests were repeated at least thrice.

## 3. Results and Discussion

### 3.1. Macrostructural and Microstructural Analyses

**Figure 3:**
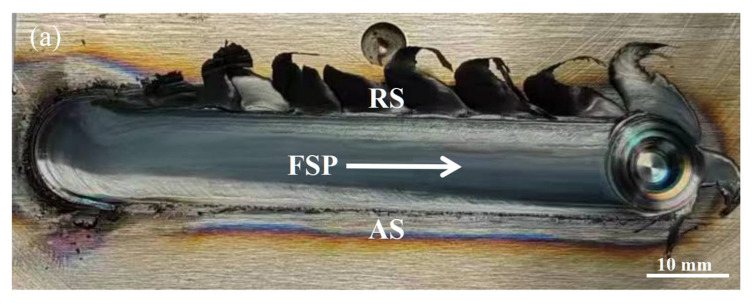

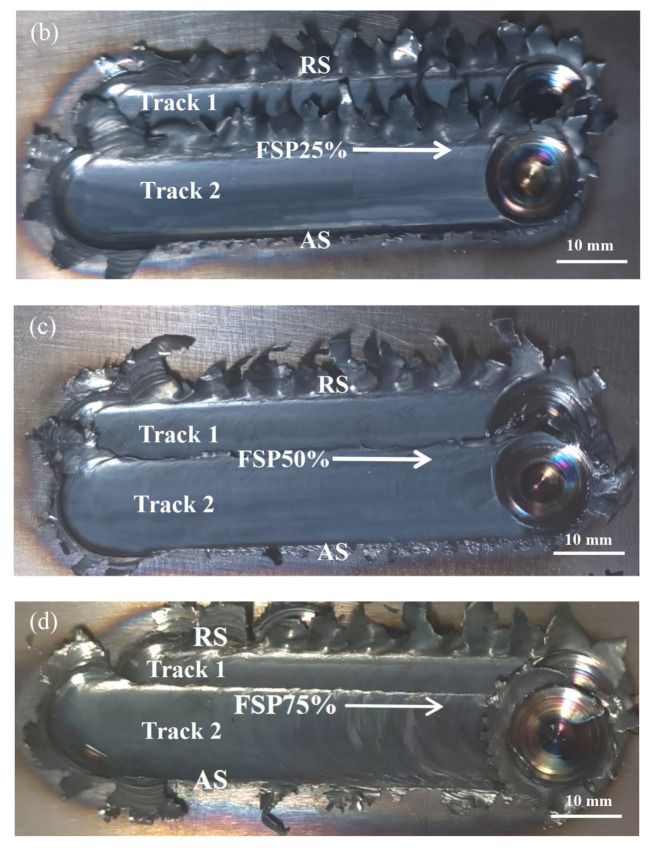
Surface morphology of (**a**) single-pass FSPed M2, and multiple-pass FSPed M2 with ORs of (**b**) FSP25%, (**c**) FSP50%, and (**d**) FSP75%.

**Figure 4:**
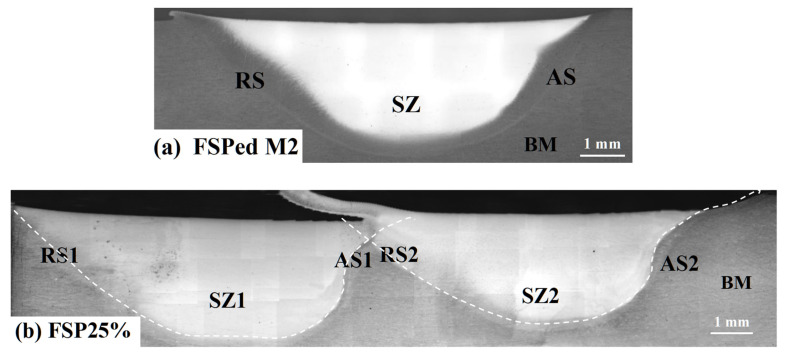

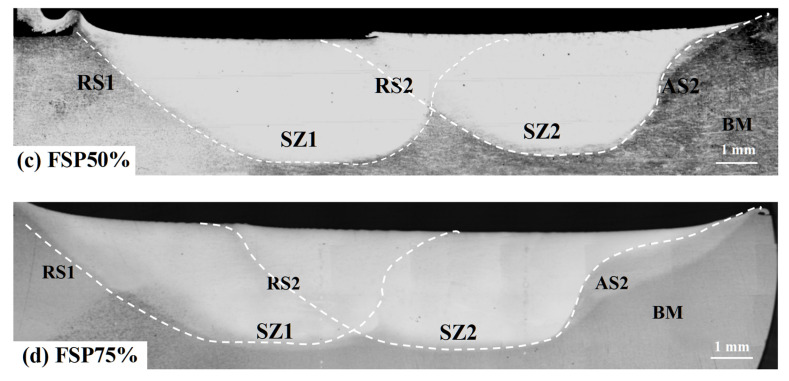
Optical micrographs of the cross-section of (**a**) single-pass FSPed M2 and multiple-pass FSPed M2: (**b**) FSP25%, (**c**) FSP50%, (**d**) FSP75%. The white dashlines distinguish the first and second passses.

Figure 3 presents the surface morphology of the single-pass and multiple-pass FSPed M2 with different ORs (25%, 50%, and 75%). After the first pass was completed, a second pass was performed along the same direction, and its RS overlapped on the AS of the first pass. Gandra et al. [27] investigated the overlapping direction of multiple-pass FSP of AA5083-H111 alloy and found that overlapping by the RS could produce smoother surfaces, while overlapping by the AS resulted in more uniform layer thickness. In addition, Liu et al. [10] reported that inappropriate FSP parameters on hypereutectic Al-Si alloy could lead to tunnels and cavities. Those defects often appeared at the bottom and AS of the stir zone (SZ). Since the rate of material flow at the AS is higher than that at the RS, the material accumulates at the RS, while the material in the AS cannot be filled in time, resulting in forming such defects. Pan et al. [23] also reported that those defects can be eliminated through multiple-pass overlapping by AS for the FSPed AISI 420. Figure 4 shows the cross-sections of the single-pass FSPed M2 and the multiple-pass FSPed M2 with different ORs. The single-pass FSPed region exhibits a width of approximately 14.5 mm and a depth of 3.8 mm. During FSP, the tangential component and transverse velocity at the RS of the SZ are opposite, leading to reduced frictional forces, lower thermal input, and diminished plastic deformation [28]. In contrast, on the advancing side (AS), the tangential and transverse velocities are in the same direction, which intensifies the thermal and mechanical effects. Avila et al. [29] reported that the peak temperature attained on the AS is notably higher than that on the RS. The FSPed region presents a basin-like shape, and the RS and AS are not symmetrical.

For the multiple-pass FSPed M2, the width and depth of the FSPed regions vary with different ORs (Figure 4). Pan et al. [23] reported that different ORs for the multiple-pass FSPed 420 greatly affected the overlapped area and hardened depth of the FSPed region. In the present study, the largest overlapped region (at OR of 75%) produced the most uniform depth in the FSPed region.

**Figure 5:**
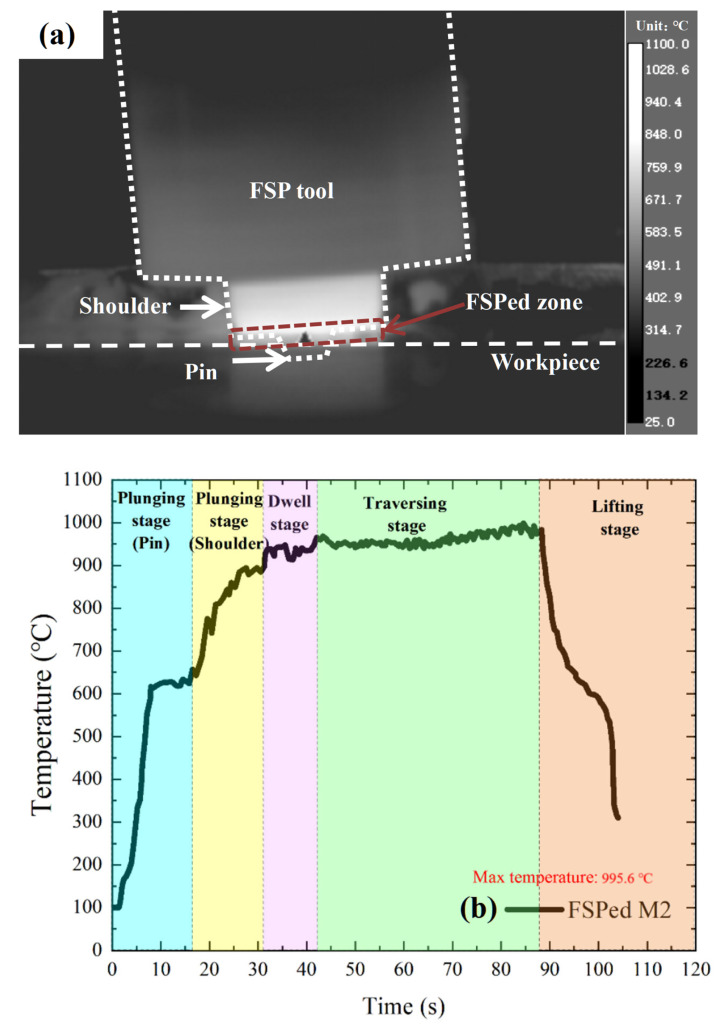
(**a**) Typical thermal image of the entire field of view and (**b**) temperature profile of FSPed M2.

Based on the calculated phase diagram of M2 [30], AR M2 consists mainly of α-ferrite and carbides (MC, M_23_C_6_ and M_6_C). For the QT M2, it contains a matrix of tempered martensite dispersed with carbides (M_6_C and MC). During FSP, a non-contact infrared camera was used to monitor the temperature evolution of M2. The infrared thermal images of the mechtrode and workpiece (the single-pass FSPed M2) captured during FSP are illustrated in Figure 5a. The temperature profile was recorded at the interface between the shoulder and the workpiece as depicted in Figure 5b. The infrared thermography was recorded using a thermal infrared imaging camera (FLIR A655sc, 640 × 480 pixels, bandwidth range of 7.5–14 μm, noise equivalent temperature difference (NETD) < 30 mK), with the emissivity set to 0.95 by default, a measurement distance of about 0.5 m, the reflection temperature of 25 °C, and an acquisition frequency of 50 Hz. Additionally, the thermal data were analyzed using FLIR ResearchIR software (version 4.40, Teledyne FLIR LLC, Wilsonville, OR, USA). Five distinct stages can be distinguished including the following: plunging stage of the pin, plunging stage of the shoulder, dwell stage, traversing stage, and lifting stage. In the plunging stage of the pin, frictional interaction between the rotating pin and the workpiece, along with plastic deformation, generated heat, and the temperature rose to 630 °C. In the plunging stage of the shoulder, as the plunging depth increased, the shoulder began to contact the workpiece and the friction between them further heated up to 950 °C. During the dwelling stage, there was a slight increase in temperature. In the traversing stage, the temperature became steady due to a balance between the heat generation and loss. The peak temperature at this stage was about 995.6 °C. During the final lifting stage, the mechtrode gradually lifted up from the workpiece and began a rapid temperature drop.

**Figure 6:**
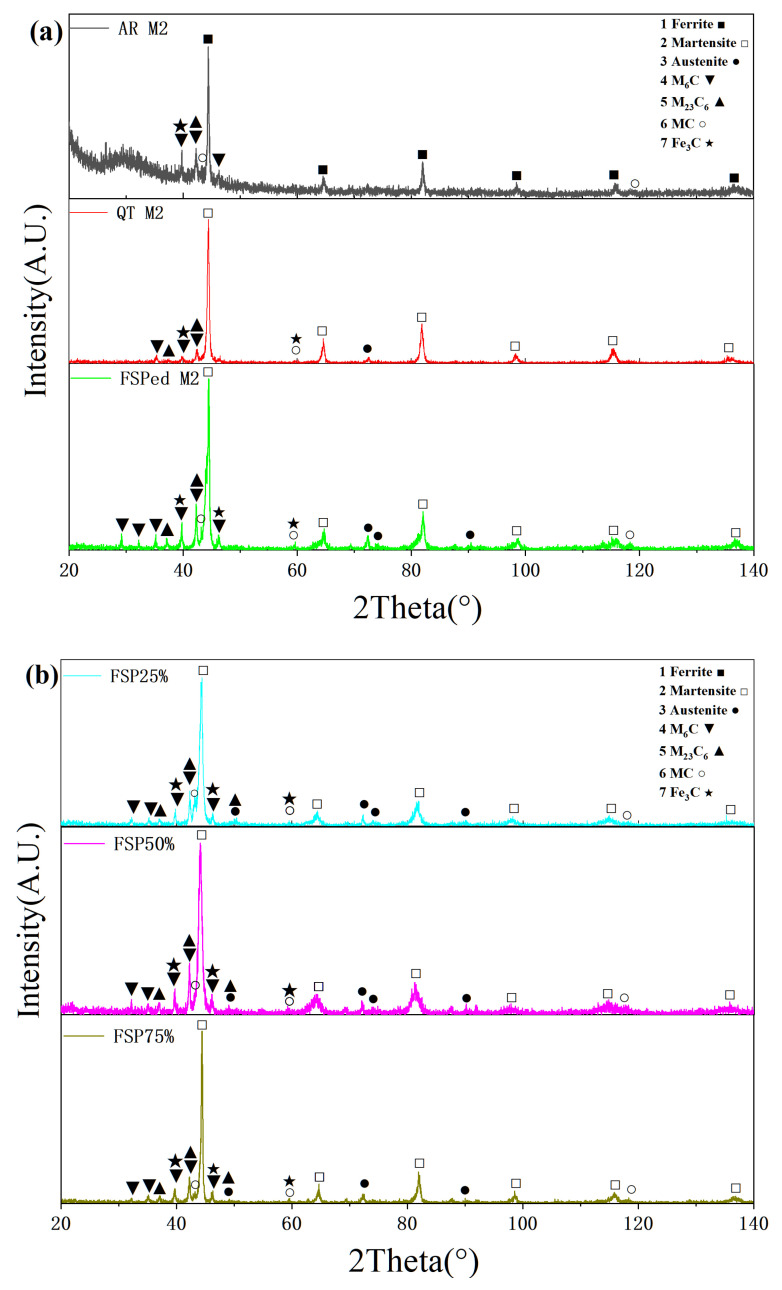
X-ray diffraction spectra of (**a**) AR M2, QT M2, and single-pass FSPed M2, and (**b**) multiple-pass FSPed M2 (FSP25%, FSP50%, and FSP75%).

**Figure 7:**
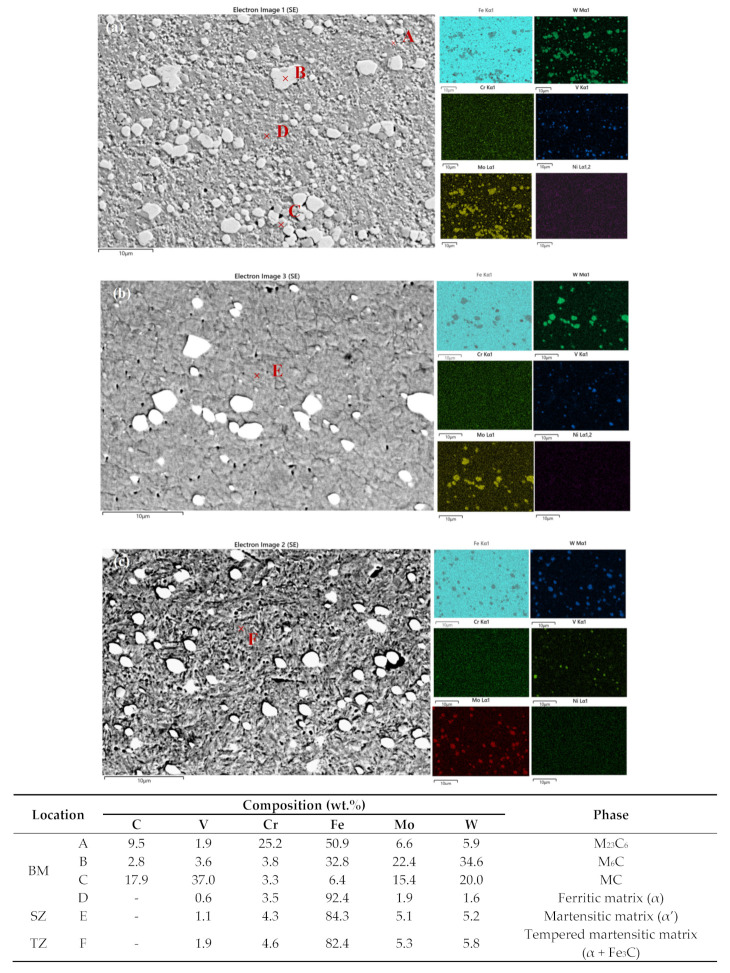
SEM micrographs, EDS maps, and chemical compositions of the phases/matrix of (**a**) BM, (**b**) OZ, and (**c**) TZ for multiple-pass FSPed M2 (FSP75%).

Figure 6 presents the XRD patterns of AR M2, QT M2, single-pass, and multiple-pass FSPed M2. According to standard JCPDS cards, the AR M2 is primarily composed of a ferritic matrix (α, BCC, JCPDS No. 06-0696), whereas the QT M2, single-pass and multiple-pass FSPed M2 are dominated by a martensitic matrix (α′, BCT, JCPDS No. 34-0396) with minor content of retained austenite (γ, FCC, JCPDS No. 33-0397). The content of austenite in all FSPed M2 seems to be slightly higher than that in QT M2 because the intensity and amount of austenite peaks (50.2°, 72.4°, 74.1° and 90.1°) in the former are higher. In addition, the secondary phases including M_6_C, M_23_C_6_, MC, and Fe_3_C are identified in all specimens. In the 2θ range of 30–50°, the single-pass and multiple-pass FSPed M2 exhibit more additional diffraction peaks assigned to various carbides [31]. These low-intensity peaks are attributed to fragmentation of coarse carbides during FSP, which promotes the formation of finer and more numerous carbides with low peak intensities. The full width at half maximum observed in the FSPed specimens also reflects grain refinement and lattice strain induced by severe plastic deformation and dynamic recrystallization. A comparison between single-pass and multiple-pass FSPed specimens reveals similar phase patterns and peak positions. However, the multiple-pass samples display slightly reduced peak intensities of the carbides. This reduction suggests enhanced grain refinement and higher defect density due to repeated thermal-mechanical cycling. The SEM micrographs and EDS elemental maps (Figure 7) further corroborate the XRD findings, confirming the presence and distribution of finely dispersed carbides within the matrix.

Figure 7 displays the SEM micrographs of the typical microstructure of the BM, OZ, and tempered zone (TZ) of the multiple-pass FSPed M2 (FSP75%). Compared to the BM (Figure 7a), fewer coarse and fine carbides were sparsely distributed in the martensitic matrix of the SZ (overlapped region, Figure 7b) and the tempered martensite of the TZ (Figure 7c). The quantity and size of the carbides in the SZ and TZ of FSP75% are considerably diminished as a result of the fragmentation and dissolution of the carbides. After the FSP, those fine carbides such as M_23_C_6_ (Cr_23_C_6_) and MC (VC) were significantly dissolved, while the coarse Mo- and W-rich carbides (M_6_C) were partially fragmented and refined. From the EDS maps depicted in Figure 7, the carbides such as MC, M_23_C_6_, M_6_C were identified. V was found to be the principal alloying element in MC. M_23_C_6_ mainly consisted of Fe and Cr, while M_6_C was primarily composed of Fe, Mo, and W [32]. From Figure 7, the contents of Mo, W, V, and Cr are markedly increased within the martensitic matrix in the SZ of the multiple-pass FSPed M2 (FSP75%) compared to the AR M2.

It is noteworthy that during the multiple-pass FSP, the TZ (Figure 7c) underwent tempering in subsequent thermal cycles, resulting in the transformation of quenched martensite formed during the first FSP to tempered martensite, and more fine carbides were formed in the α-matrix. From Figure 7c, the tempered martensite presents dense needle-like and plate-like structures, and a smaller number of fine carbides of MC and M_6_C are randomly distributed in the matrix. Owing to the extremely short tempering time during the multiple-pass FSP, the precipitated sub-micron carbides cannot be clearly observed in Figure 7c. Liu et al. [33] reported that tempering of M42 HSS at 600–700 °C could lead to transformation of martensite to tempered martensite with some sub-micron carbides (< 0.3 mm) formed in the a grains, primarily nucleating at the boundaries of the a phase, as confirmed by transmission electron microscope (TEM) analysis, while Garasi [34] investigated tempering of X38CrMoV5-1 hot-work tool steel and found that the nano-sized carbides (60–100 nm) rich in Fe, Cr, and V were detected in the a-matrix. In the present study, compositional analyses of the SZ and TZ reveal an increase in the contents of Mo, W, and V in the a-matrix, as well as a significant reduction in the Fe content, which may be attributed to the formation of more Fe_3_C during the tempering [35]. The back-tempering effect in the multiple-pass FSP has a substantial impact on the microstructure and properties of the TZ.

Figure 2 presents the location where the back-tempering phenomenon occurred in the multiple-pass FSPed M2. The purpose of multiple-pass FSP is to fabricate a large FSPed surface with uniform depth. Nevertheless, the drawback of overlapping in heat-treatable steels is the drop in hardness owing to back-tempering near the overlapped regions of the two successive passes [36]. This phenomenon is due to the interaction between the thermal field generated by friction during the second pass and the microstructure formed in the first pass (quenched martensite and retained austenite), leading to the formation of tempered martensite in the tempered zone near the RS2. The martensite is tempered at the TZ, leading to the formation of the softer tempered martensite. Giorleo et al. [37] reported the back-tempering effect during the multiple-pass laser surface treatment of AISI 1060, and the hardness of the TZ was significantly reduced to 450–500 HV_0.3_ as compared to that of the hardened zone (800–900 HV_0.3_). Similarly, in the present study, a notable hardness reduction was observed in the TZ, where the hardness dropped to around 650 HV_0.2_, compared to 900 HV_0.2_ in the SZ.

In the context of metallography, the austenitizing of HSSs followed by rapid cooling induces the formation of martensite, characterized by an abundance of supersaturated carbon and a notable dislocation density in the martensite [38]. Since carbon atoms possess a lower diffusion activation energy compared to other alloying elements, they diffuse more readily in alloy steels [39]. For conventional heat-treating of M2 HSS, the range of tempering temperature is between 540 and 580 °C, which can achieve a high hardness of 64–66 HRC and is slightly lower than the as-quenched state [40]. Wang et al. [41] investigated the effect of tempering temperature (450–650 °C) on grain refinement and transformation of martensite to tempered martensite of M2, and it was found that the alloying elements and carbon segregated at the grain boundaries, where formation of carbide subsequently occurred. For the TZ of the multiple-pass FSPed M2, the supersaturated carbon atoms in the martensite initially diffuse from the interstitial sites. Then, the transformation from the metastable martensite (BCT) to the stable ferrite (BCC) occurs. Concurrently, the diffused carbon atoms react with alloying elements to form carbide phases [42], predominantly precipitating at the retained austenite grain boundaries and the lath boundaries of the martensite [42], leading to the formation of various carbides such as M_6_C, M_23_C_6_, MC, and Fe_3_C in M2. Based on the reports of Yao et al. [35], the tempering leading to the formation of tempering martensite is as follows:

Martensite (a’) → Tempered martensite (Ferrite (a) + Carbides).

### 3.2. Hardness

**Figure 8:**
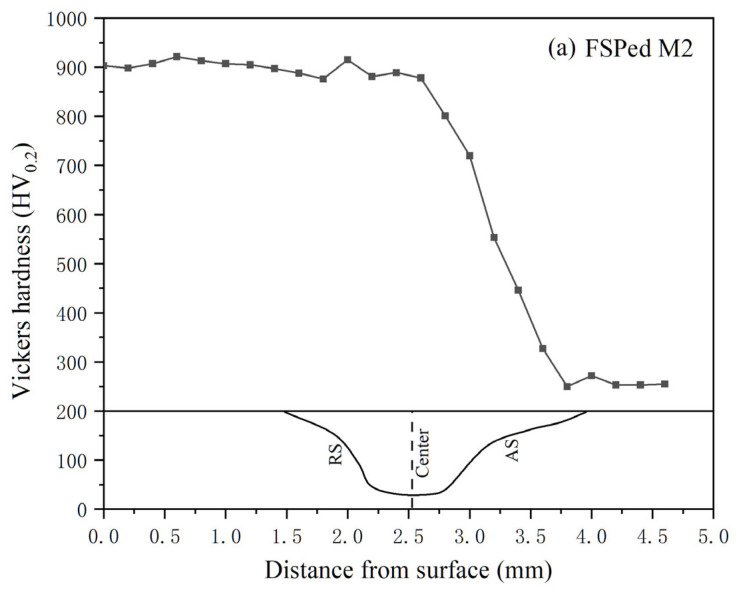

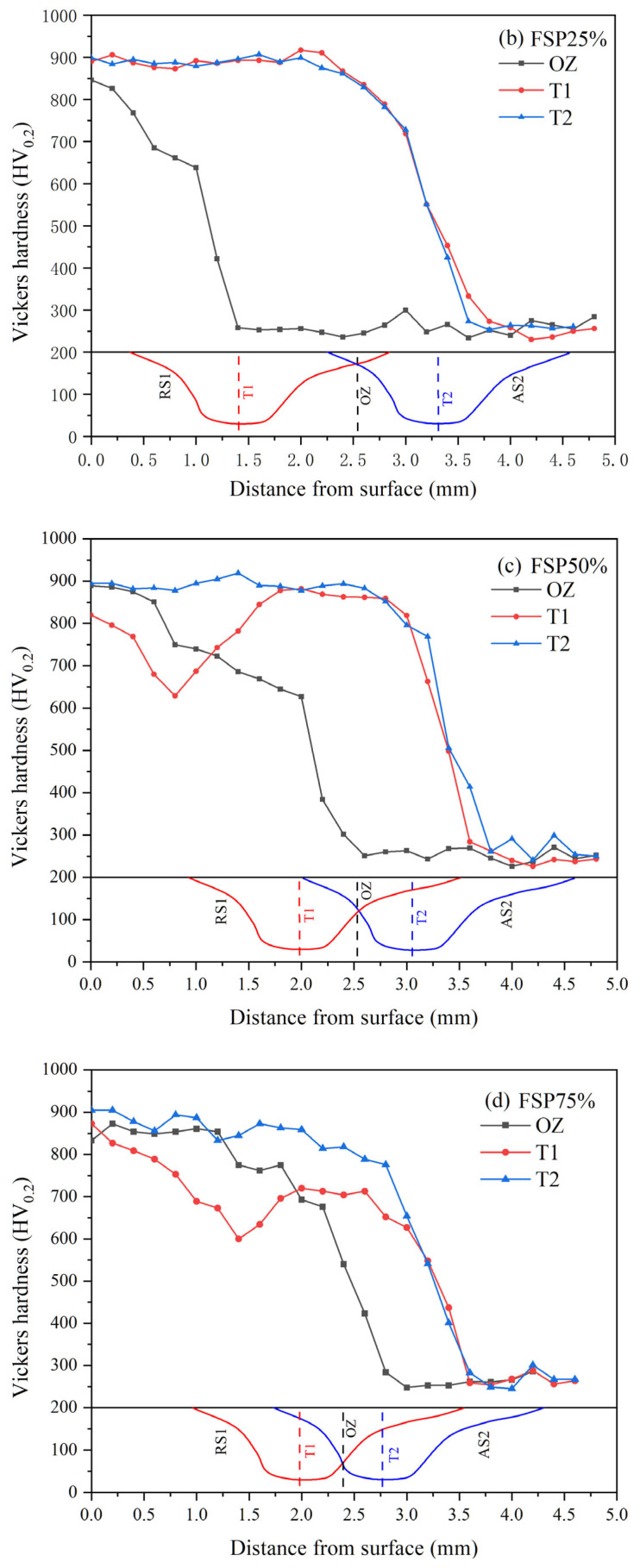
Hardness of (**a**) single-pass FSPed M2 and multiple-pass FSPed M2: (**b**) FSP25%, (**c**) FSP50%, and (**d**) FSP75% along the overlapped zone (OZ), first track (T1), and second track (T2). The figure below the hardness profiles shows the single pass or multiple passes.

Hardness profiles of the single-pass FSPed M2 and multiple-pass FSPed M2 with ORs of 25%, 50%, and 75%, measured along the centerlines of the first track (T1), second track (T2), and overlapped zone (OZ), are presented in Figure 8. The hardness profiles of the center of single-pass FSPed M2 and the T2 of all multiple-pass FSPed M2 (FSP25%, FSP50%, and FSP75%) are similar, which maintain a maximum hardness of 900 HV_0.2_ at the surface. The hardness decreases gradually along the depth from 2.5 to 3.75 mm (i.e., in the HAZ) and finally drops to a constant hardness of 260 HV_0.2_ (i.e., the hardness of BM). A decreasing trend of hardness along the depth can be observed due to the decrease in temperature and less martensite in the SZ. The non-uniform distribution and conduction of frictional heat along the depth result in temperature gradients, which in turn lead to varying the degrees of martensitic transformation across different regions.

For FSP25%, the hardness profile of T1 resembles that of T2, as the OR is relatively small and the TZ is far away from T2 (Figure 8a). However, for the hardness profiles of T1 (the red one) of FSP50% and FSP75% (Figure 8b,c), high hardness was maintained at 820–870 HV_0.2_ near the surface, followed by a sudden drop in hardness to 650 HV_0.2_ at the TZ, then increased again at the SZ. The decrease in hardness of T1 of FSP50% and FSP75% is attributed to back-tempering, specifically the influence of the thermal cycle during the second pass. This process causes the diffusion of carbon atoms in the quenched martensite in the TZ, resulting in a decrease in lattice distortion and dislocation density [43]. When the martensite with high dislocation is subjected to a high temperature, the dislocation density decreases as a result of annihilation and recovery mechanisms, and it is anticipated that the dislocation density will further decrease with increasing tempering temperature and prolonged holding time [44]. In the hardness profiles of the OZ of all multiple-pass FSPed M2, high hardness was maintained near the surface (approximately 830–900 HV_0.2_).

**Figure 9:**
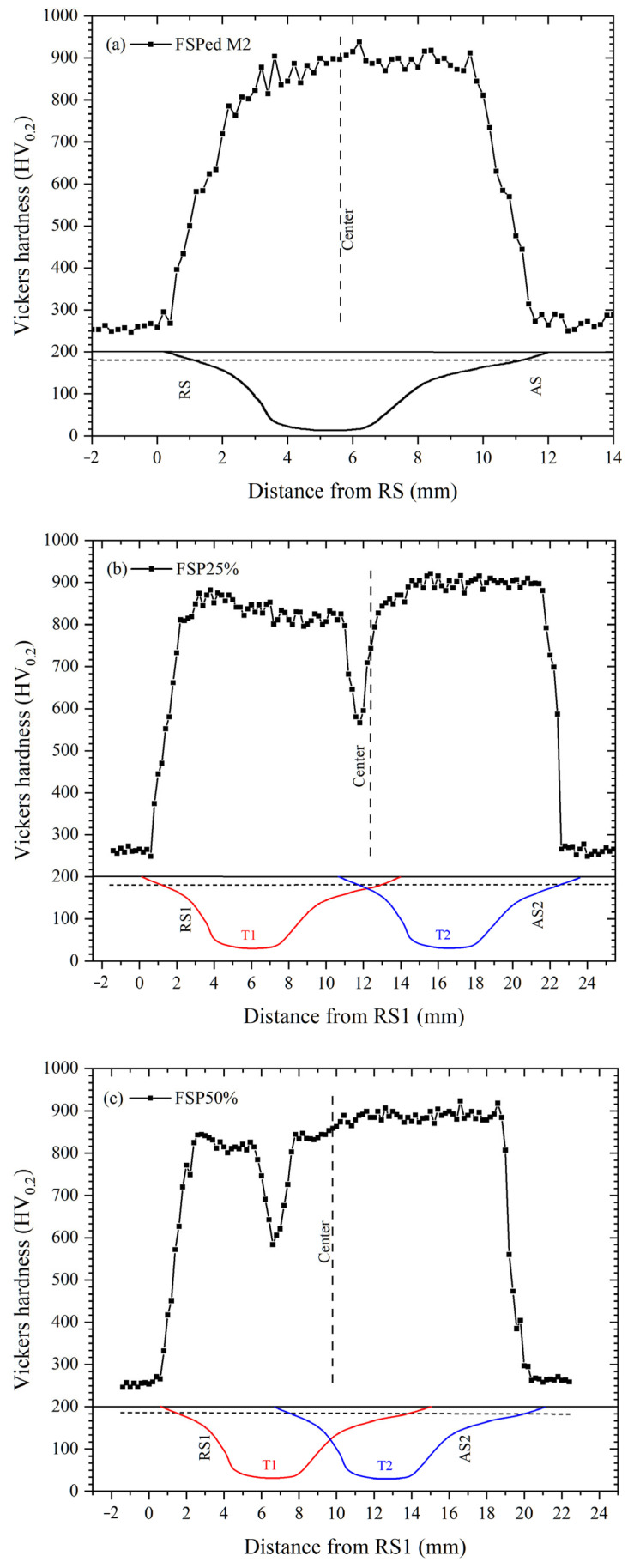

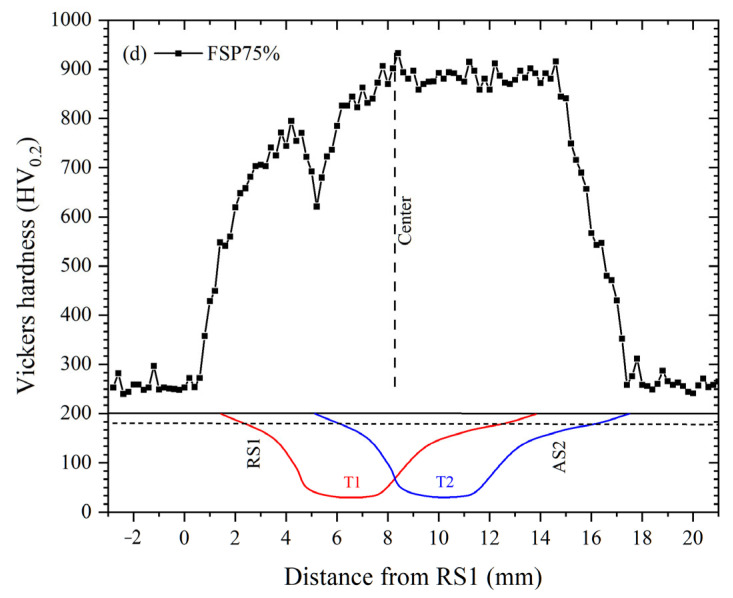
Hardness of (**a**) single-pass FSPed M2 and multiple-pass FSPed M2: (**b**) FSP25%, (**c**) FSP50%, and (**d**) FSP75% at 1 mm below the top surface across the longitudinal direction. The figure below the hardness profiles shows the single pass or multiple passes.

As reported in the previous work [2], the longitudinal hardness distribution of single-pass FSPed M2 was found to be non-uniform, mainly maintaining at 800–900 HV_0.2_ in the SZ. The highest hardness appeared in the SZ, whereas a significant reduction was observed in the thermo-mechanically affected zone (TMAZ) and the heat-affected zone (HAZ) on both AS and RS. The hardness reduction was attributed to temperature gradients, incomplete martensitic transformation, and heterogeneous microstructures in these zones. In the present study, Figure 9 illustrates the longitudinal hardness profiles of the single-pass FSPed M2 and multiple-pass FSPed M2 with different ORs, measured at a depth of 1 mm below the top surface. A sharp decrease in hardness to about 650 HV_0.2_ is observed on the left side of the OZ (the RS of the T2) owing to back-tempering [36].

The Hollomon–Jaffe parameter, known as the tempering parameter, describes the softening effect on steel in a tempering period. It can be calculated as follows [45]:

H = T [C + log(t)](2)

where H is the hardness, T is the temperature, C is a constant mainly related to the compositions of the steels, and t is time. It is evident that the identical alteration in hardness can be attained either by subjecting the material to low temperature for an extended duration or by exposing it to high temperature for a short period of time. During FSP, it is mainly the instantaneous frictional heat generation that can cause a high temperature, although the holding time is very short, possibly being only a few seconds. The hardness in the TZ dropped to as low as 650 HV_0.2_ approximately, indicating that tempering can lead to an uneven surface, decrease in surface hardness, and reduced wear resistance [36].

### 3.3. Wear Behavior

**Figure 10:**
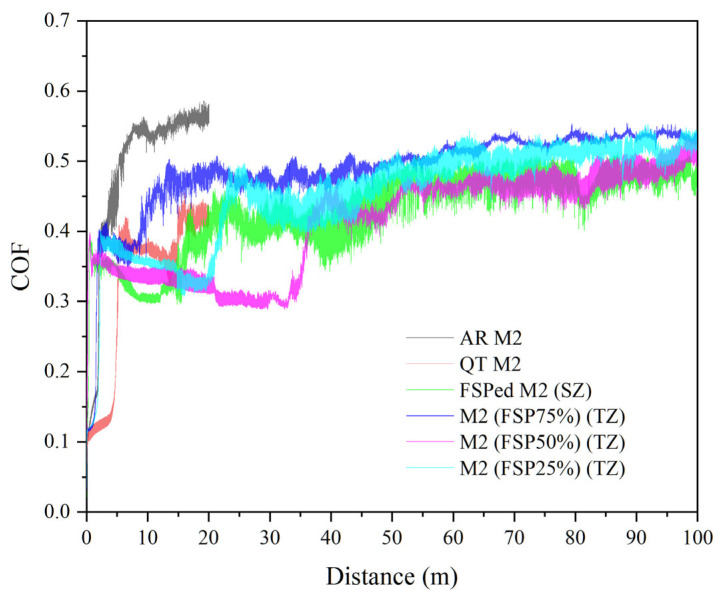
Plot of COF against distance for AR M2, QT M2, SZ of single-pass FSPed M2 and TZ of multiple-pass FSPed M2 (FSP25%, FSP50%, and FSP75%).

Figure 10 shows the plot of coefficient of friction (COF) vs. sliding distance of the AR M2, QT M2, and SZ of the single-pass FSPed M2, and TZ of the multiple-pass FSPed M2 (FSP25%, FSP50%, and FSP75%). The initial COF increases rapidly, which was mainly due to the transition from static friction to dynamic friction [46]. The COF of the AR M2 increased to about 0.55 in the initial stage and the wear loss was substantial, so the wear test was terminated after sliding for 20 m. In the initial stage (slid for 20 m), the COF of the QT M2, SZ of the single-pass FSPed M2, and TZ of the multiple-pass FSPed M2 (0.3–0.45) were significantly lower than that of the AR M2. This lower COF was mainly attributed to the presence of hard martensite in the QT M2 and the SZ of the single-pass FSPed M2, as well as the presence of tempered martensite in the TZ of the multiple-pass FSPed M2. The ability to resist plastic deformation of the hard martensite and tempered martensite was higher in the initial stage. Following the running-in period during wear, the stable COF values of the TZ of the multiple-pass FSPed specimens (FSP25%: 0.50, FSP50%: 0.47, and FSP75%: 0.50) were slightly higher than those of the SZ of single-pass FSPed M2 (0.45). The reduced hardness in the TZ promotes greater material adhesion and plowing effects during sliding, thereby elevating COF [47]. The initial increase in COF corresponds to the running-in period where surface asperities engage, while subsequent stabilization defines the steady-state regime.

**Figure 11:**
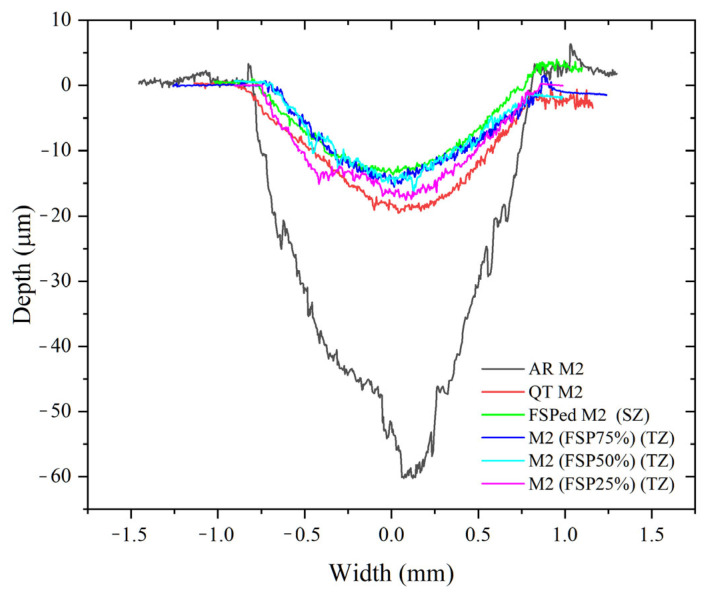
Surface profiles of wear tracks of SZ of single-pass FSPed M2, TZ of multiple-pass FSP specimens (FSP75%, FSP50%, and FSP25%), QT M2, and AR M2.

Figure 11 shows the wear profiles of various specimens. The average hardness, COF, the calculated values of the wear volume, wear rate, and normalized wear resistance of all specimens are shown in Table 3. The COF of the TZ of the multiple-pass FSPed M2 is higher than that of single-pass FSPed M2. The average hardness of the TZ of the multiple-pass FSPed M2 is also lower than the SZ of the FSPed M2, which indicates a higher wear volume and wear rate of the former. The wear rates of the TZ of the multiple-pass FSPed M2 (FSP25%: 1.40 × 10^−5^ mm^3^/N·m, FSP50%: 1.20 × 10^−5^ mm^3^/N·m, and FSP75%: 1.00 × 10^−5^ mm^3^/N·m) are all higher than that of the SZ of the single-pass FSPed M2 (0.75 × 10^−5^ mm^3^/N·m), indicating lower wear resistance of the TZ. Nevertheless, the TZ of the multiple-pass FSPed M2 still exhibits significantly higher wear resistance than the AR M2 (10.50 × 10^−5^ mm^3^/N·m) and QT M2 (3.00 × 10^−5^ mm^3^/N·m). It is attributed to grain refinement and the uniform distribution of carbides achieved through the thermo-mechanical stirring effect during FSP, as well as the higher martensite content in the FSPed specimens (81.8%) compared to QT M2 (75.7%) [2]. The normalized wear resistances of the SZ and TZ of the FSPed specimens are significantly improved and superior to those of AR M2 and QT M2. Specifically, the normalized wear resistance of the SZ of the FSPed M2 shows a 14-fold improvement, while those of the multiple-pass FSPed M2 (FSP25%, FSP50%, and FSP75%) exhibit improvements of 7.5, 8.8, and 10.5 times, respectively.

**Figure 12:**
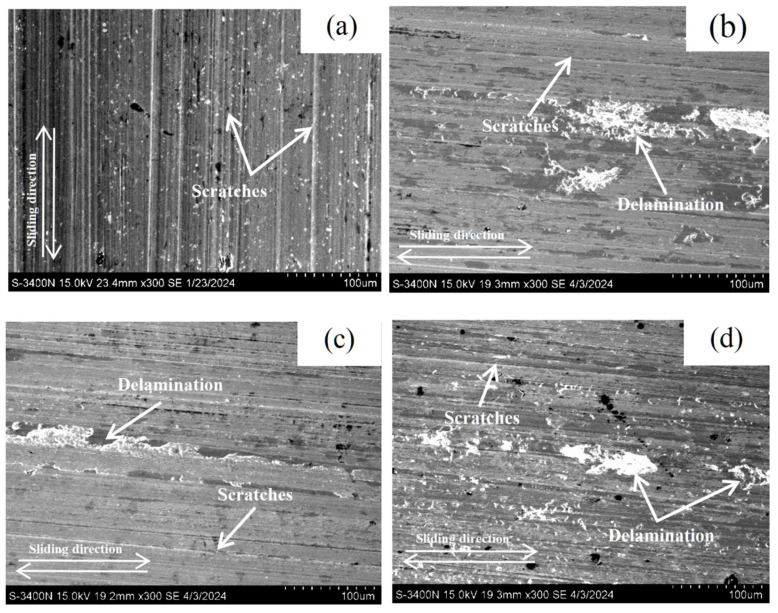
SEM micrographs of worn surfaces of (**a**) SZ of FSPed M2, and TZ of multiple-pass FSPed M2: (**b**) FSP25%, (**c**) FSP50%, and (**d**) FSP75%.

It was reported that adhesive wear (removal of material through plastic deformation) and abrasive wear were the main wear mechanisms for the AR M2 and QT M2, while the single-pass FSPed M2 primarily exhibited abrasive wear [2]. To further investigate the wear mechanism of the multiple-pass FSPed M2 (FSP25%, FSP50%, and FSP75%), SEM micrographs of the worn surfaces are shown in Figure 12. For the single-pass FSPed M2, a few shallow scratches are observed, indicating a lower degree of wear damage (Figure 12a). On the worn surfaces of the TZ of the multiple-pass FSPed specimens, grooves are visible along with localized delamination and wear debris. These features provide evidence of the micro-welded junctions formed at the contact interfaces during the sliding wear, which subsequently fractured under applied load [48]. This resulted in localized material detachment and the occurrence of adhesive wear. The increase in wear volume of the TZ of the multiple-pass FSPed M2 is attributed to the reduction in hardness caused by back-tempering, which increases the ductility of the material. These observations are consistent with the delamination and spalling features observed on the worn surface of the multiple-pass FSPed M2, indicating that the dominant wear mechanism is abrasive wear accompanied by a certain degree of adhesive wear.

### 3.4. Corrosion Behavior

**Figure 13:**
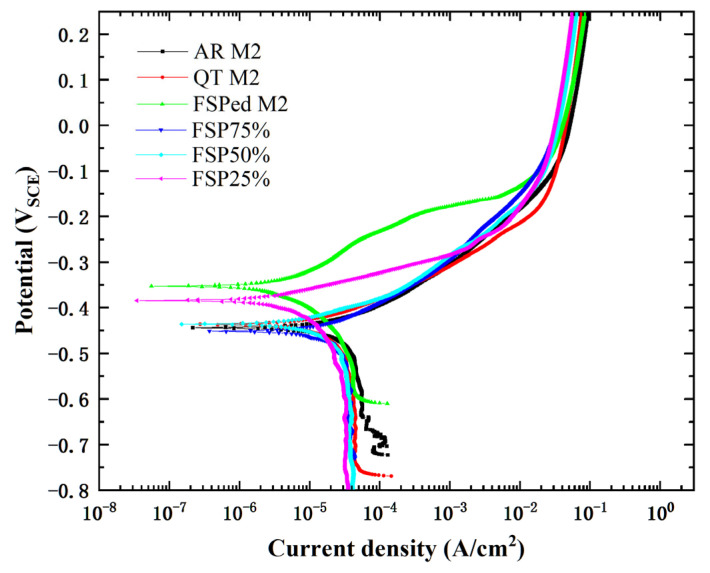
PD curves of FSPed M2, FSP25%, FSP50% and FSP75%, AR M2 and QT M2 in 3.5% NaCl solution at 25 °C.

Figure 13 displays the PD curves of the single-pass FSPed M2, multiple-pass FSPed M2 (FSP25%, FSP50%, and FSP75%), AR M2, and QT M2 in 3.5% NaCl at 25 °C. The corresponding E_corr_ and I_corr_ are depicted in Table 4. FSP generally enhances corrosion resistance by enriching the martensite matrix with passivating elements (W, V, Mo, and Cr) which promote stable protective films [2,49]. The stable passivation film was formed on the surface to protect the matrix and is significantly better than that of the AR M2 and QT M2. Generally, a nobler corrosion potential (E_corr_) and a lower corrosion current density (I_corr_) are indicative of higher corrosion resistance [49].

In contrast to the single-pass FSPed M2, the overall corrosion properties of the multiple-pass FSPed M2 become worse, as shown in Figure 13 and Table 4. The E_corr_ and I_corr_ of FSP25% are −397.6 mV_SCE_ and 9.5 μA/cm^2^, respectively, while the E_corr_ and I_corr_ of FSP50% are −424.4 mV_SCE_ and 14.7 μA/cm^2^, respectively. Among them, the corrosion resistance of FSP75% is the lowest with E_corr_ of −440.9 mV_SCE_ and I_corr_ of 17.1 μA/cm^2^. The deterioration of corrosion resistance is attributed to presence of more carbides including M_6_C, M_23_C_6_, MC, and Fe_3_C in the TZ [33,50], as well as the depletion of passivating alloying elements (W, Mo, V, and Cr) at the vicinity of the a-matrix, leading to the formation of active sites. As a result, a continuous and dense protective layer fails to form at such locations. Moreover, the increased number of active sites around the carbides further disrupts the uniformity of the passivation layer and deteriorates the corrosion resistance of the multiple-pass FSPed M2. Jurci et al. [51] compared the sub-zero treatments and tempering processes applied to Vanadis 6 tool steel and found that the tempered specimens were more anodic and had inferior corrosion resistance. This degradation can be attributed to the transformation of the martensitic matrix into tempered martensite after tempering, and the precipitation of more Cr-rich carbides such as M_7_C_3_ in the matrix introduced additional active sites for corrosion, which reduced the amount of Cr atoms in solid solution within the matrix and further contributed to the deterioration in corrosion performance.

**Figure 14:**
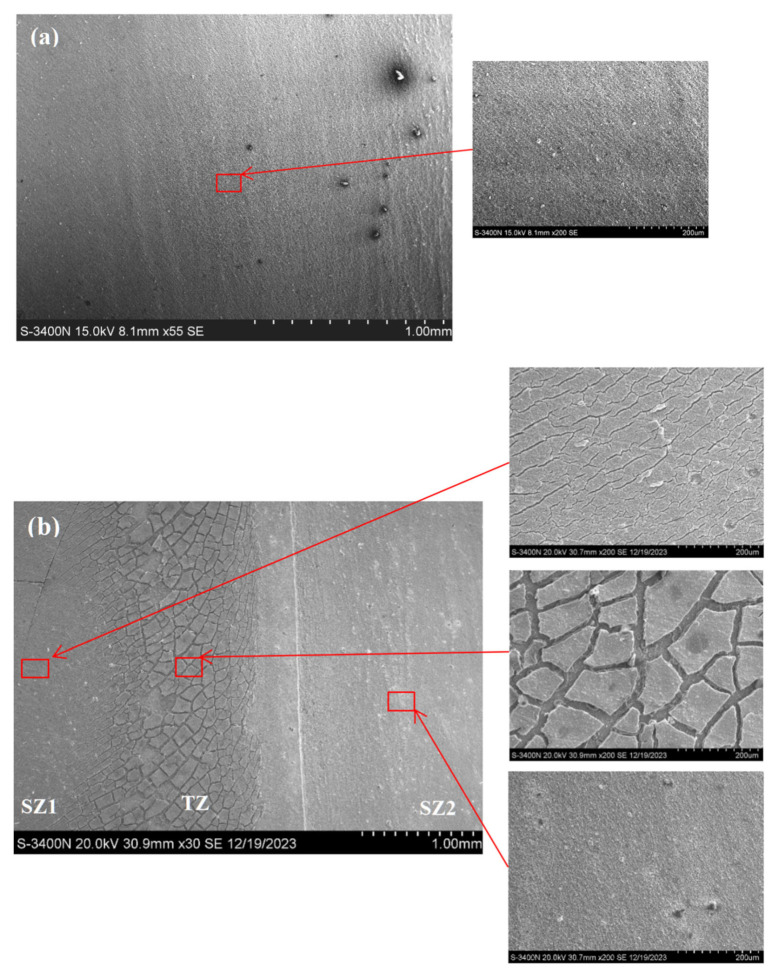

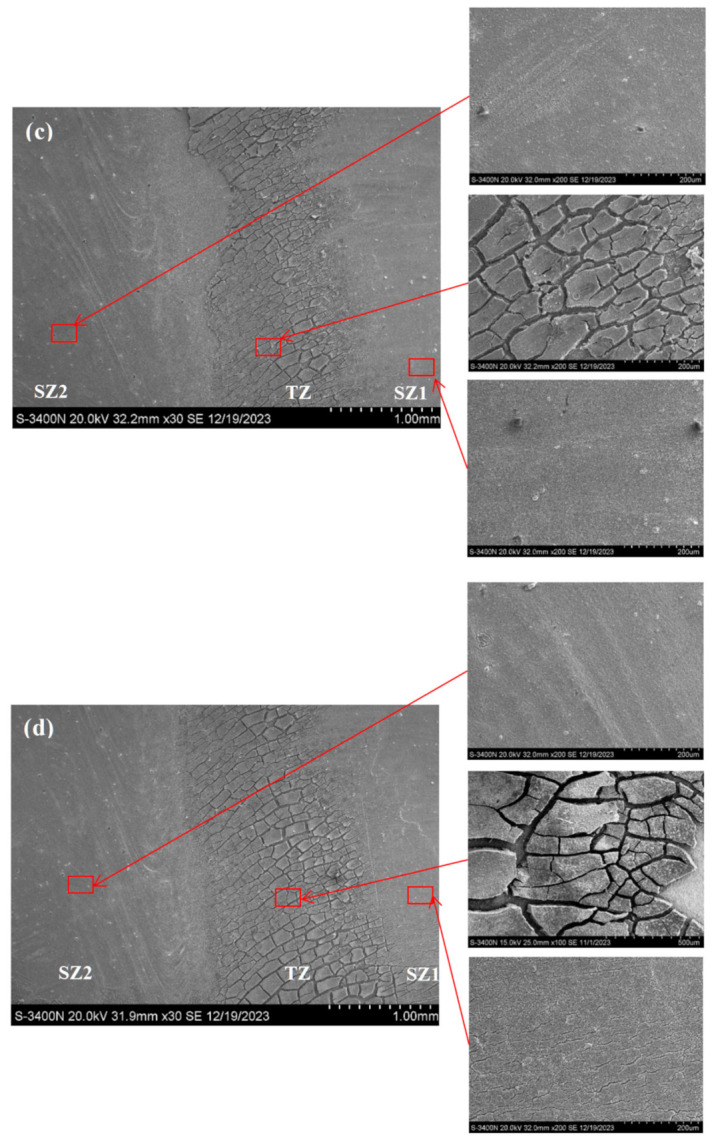
Corrosion morphology of (**a**) single-pass FSPed M2, (**b**) FSP25%, (**c**) FSP50%, and (**d**) FSP75% after PD tests in 3.5 wt.% NaCl solution.

**Figure 15:**
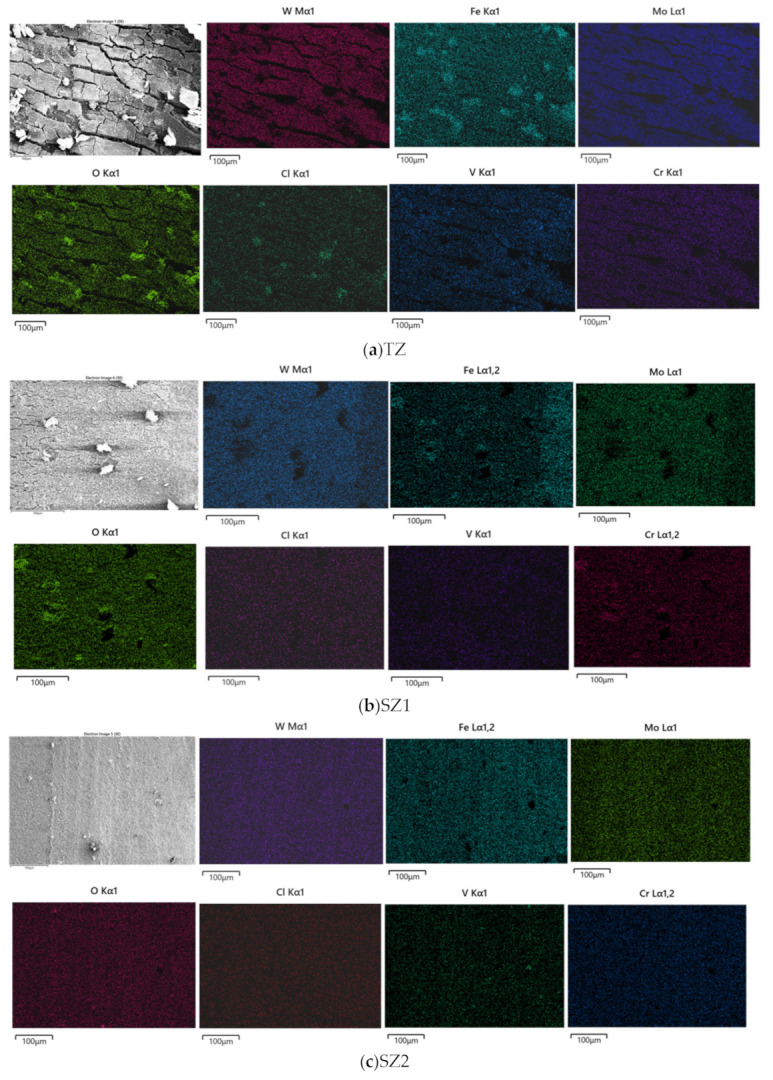
EDS analysis of corroded surfaces of (**a**) TZ, (**b**) SZ1, and (**c**) SZ2 of FSP75% after PD test.

Figure 14 illustrates the corrosion morphologies of the single-pass FSPed M2, and the multi-pass FSPed M2 (FSP25%, FSP50%, and FSP75%) after the PD tests. It is apparent that the corrosion morphologies in the TZ of the multiple-pass FSPed M2 are obviously different from the SZ of the single-pass FSPed M2. The mud-crack structures can be clearly observed in the TZ of the multiple-pass FSPed M2 (Figure 14b–d) while the surface of the single-pass FSPed M2 is covered by a relatively uniform oxide layer (Figure 14a), which provides effective passivation protection. Ripoll et al. [52] investigated the corrosion behavior of laser-hardened HSSs and reported that untreated HSSs exhibited a discontinuous corrosion layer with characteristic mud-crack structures after the polarization test. EDS analysis further elucidated that this corrosion layer primarily consisted of iron oxides, with the presence of alloying elements and carbon as minor compositions. Figure 15 presents EDS maps of the corroded surfaces of the TZ, SZ1, and SZ2 of the FSP75%. The compositions of the mud-crack structure in the TZ mainly contain Fe and O, along with some alloying elements including Mo, W, V, and Cr. Corrosion products of iron oxides and chlorides were also detected on the corroded surface. On the contrary, the corroded surface of SZ1 and SZ2 are milder with fewer corrosion products, with more homogeneously distributed alloying elements. Compared to the single-pass FSPed M2, the multiple-pass FSPed M2 specimens exhibit the larger mud-crack structure area in the corrosion test region, aligning with PD results. Notably, the I_corr_ values for FSP25%, FSP50%, and FSP75% are higher, indicating the higher corrosion rates.

**Figure 16:**
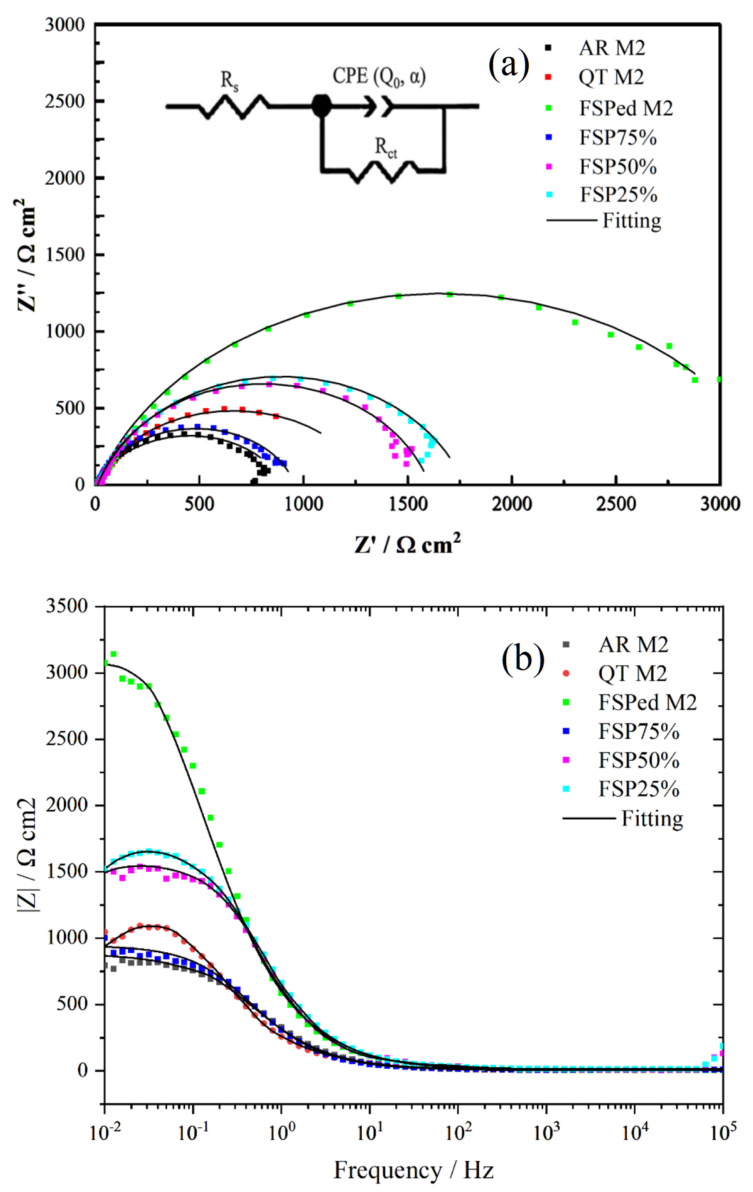

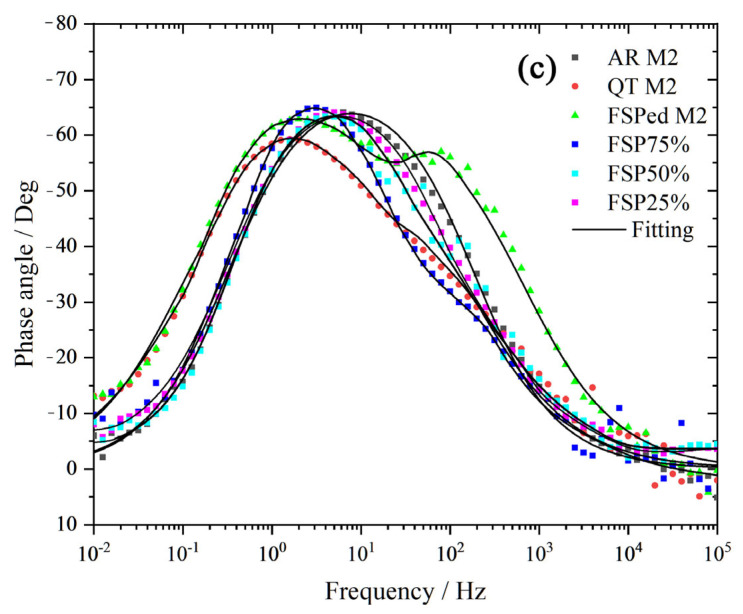
(**a**) Nyquist plots and (**b**,**c**) Bode plots of EIS results of FSPed M2, FSP25%, FSP50% and FSP75%, AR M2 (annealed) and QT M2 (quenched and tempered).

EIS curves and the equivalent circuit model of the single-pass and multiple-pass FSPed M2, AR M2, and QT M2 in 3.5 wt% NaCl solution (open to air) at 25 °C are shown in Figure 16a. In this model, R_s_ symbolizes the solution resistance, which is connected in series with two elements arranged in parallel. R_ct_ is indicative of the charge transfer resistance. The constant phase element (CPE), which reflects the characteristics of the double layer, is characterized by an exponent n. The expression for the CPE is given by Z_CPE_ = (Q(jω)α)^−1^, where Q is the constant associated with the CPE, j is, $\sqrt{-1}$ ω denotes the angular frequency in rad/s, and α is the CPE’s dispersion coefficient. The α value ranges between −1 (representing pure inductive behavior) and +1 (representing pure capacitive behavior).

EIS results are fitted by the equivalent circuit model (Figure 16a) for various samples, as displayed in Table 5. The larger R_ct_ means higher corrosion resistance. In the Nyquist plots, a consistent observation is the presence of a single capacitive arc, indicative of a uniform electrochemical behavior in 3.5 wt% NaCl solution (open to air) at 25 °C. The R_ct_ of the single-pass FSPed M2 is larger than that of the AR and QT M2. The values of R_ct_ of the multiple-pass FSPed M2 (FSP25%, FSP50%, and FSP75%) are significantly lower (1957.55, 1805.98, and 937.85 Ω·cm^2^ respectively). Among them, the R_ct_ of FSP75% is slightly smaller than QT M2, which means that the corrosion resistance (CR) of FSP75% is slightly inferior to QT M2.

Figure 16b,c depicts the Bode plots of EIS results of various specimens, respectively. The Bode plots illustrate that at low frequencies of 10^−2^ to 10^−1^ Hz, AR M2 exhibits the lowest impedance value, whereas the single-pass FSPed M2 demonstrates the highest impedance value, approximately 3074.6 Ω·cm^2^. However, the impedance values of multiple-pass FSPed M2 slightly decrease, with values for FSP25% of 1657.2 Ω·cm^2^, FSP50% of 1522.3 Ω·cm^2^, and FSP75% of 923.9 Ω·cm^2^. The reduction in impedance values of the multiple-pass FSPed M2 is primarily attributed to the formation of tempered martensite and precipitated carbides in the TZ leading to a decrease in corrosion resistance [53]. The above findings of the Bode plots of impedance in Figure 16b are basically consistent with those of the polarization tests. From the phase angle plots, it is evident that within the frequency range of 10^−2^ to 10^5^ Hz, the majority of specimens exhibit a similar minimum phase angle. Notably, the FSPed M2 shows two phase angle peaks at frequencies 10° and 10^2^, with values of 63.88° and 57.05°, respectively.

## 4. Conclusions

The microstructure observed in the stir zones (SZ) of the multiple-pass FSPed M2 with different overlapping ratios (OR) of 25%, 50%, and 75% is similar to that of the single-pass FSPed M2, consisting of martensite, retained austenite, and carbides (M_6_C, M_23_C_6_, and MC). However, in the tempered zone (TZ) for the multiple-pass FSPed M2, local back-tempering caused transformation of the hard martensite to the less hard tempered martensite.

The tempering phenomenon occurred near the overlapping of two successive tracks of M2 (around the RS of the second tracks), resulting in a significant decrease in hardness in the TZ, dropping from a peak of 900 HV_0.2_ to as low as around 650 HV_0.2_.

FSP markedly improved the wear resistance of M2 steel. The SZ of single-pass FSPed M2 exhibited the highest hardness, lowest COF (0.45), and wear rate (0.75 × 10^−5^ mm^3^/N·m), achieving a 14-fold increase in wear resistance. In contrast, the TZ of multiple-pass specimens showed reduced hardness and higher COF (0.47–0.50), with wear rates of 1.00–1.40 × 10^−5^ mm^3^/N·m, yet still demonstrated 7.5–10.5 times greater wear resistance than AR M2, also better than that of QT M2.

The results of electrochemical tests reveal that multiple-pass FSPed M2 exhibits inferior corrosion resistance compared to single-pass FSPed M2 (E_corr_: −397.6 mV_SCE_ and I_corr_: 4.5 μA/cm^2^). With increasing OR (FSP25–FSP75%), E_corr_ shifts in the active direction (−397.6 to −440.9 mV_SCE_) and I_corr_ increases (9.5 to 17.1 μA/cm^2^), indicating accelerated corrosion. EIS analysis confirms this trend: R_ct_ values of FSP25%, FSP50%, and FSP75% (1957.55, 1805.98, and 937.85 Ω·cm^2^ respectively) are markedly lower than single-pass FSPed M2 (3647.03 Ω·cm^2^). Mud-crack corrosion morphologies and non-uniform passivation layers in the TZ, which could be attributed to more carbide precipitation in the TZ, a reduction in passivating elements, and an increase in active corrosion sites near those carbides, collectively contribute to diminished corrosion resistance.

During multiple-pass FSP surface strengthening of heat-treatable steels such as M2 high-speed steel, localized back-tempering effect can induce microstructural inhomogeneity, reducing both mechanical properties and corrosion resistance. This effect primarily arises from the interaction between the thermal cycle of the second pass and the quenched martensite formed in the first pass, leading to the formation of tempered martensite near the RS2 of the OZ. Controlling the thermal field during FSP is critical to mitigating this tempering effect. Reducing rotational speed while increasing traverse speed can lower frictional heat generation, thereby decreasing the overall heat input and processing temperature. Alternatively, implementing auxiliary cooling methods during FSP, such as water or liquid nitrogen, can increase the cooling rate, limit the temperature and extent of the thermal field, and suppress tempering-induced softening in the HAZ. These strategies offer promising avenues for optimizing multiple-pass FSP and enhancing the mechanical and corrosion performance of treated heat-treatable steels in industrial settings.

## Figures and Tables

**Table 1:** Chemical compositions of M2 (wt%).

Composition (wt%)	Fe	W	Mo	Cr	V	Mn	Ni	Si	C
M2	Bal.	8.52	6.52	3.33	2.13	0.31	0.29	0.23	0.90

**Table 2:** FSP parameters used in this study.

FSP parameters	Values
Rotation speed	600 rpm
Traveling speed	100 mm/min
Plunged depth	4.1 mm
Plunged speed	5 mm/min
Z-axis lifting speed	50 mm/min
Z-axis lifting height	20 mm
Plunged delay time	7 s
Lift delay time	2 s
Overlapping ratios	25%, 50%, and 75%

**Table 3:** Average hardness, COF, wear volume, wear rate, and normalized wear resistance of FSPed M2 (SZ), FSP25% (TZ), FSP50% (TZ), and FSP75% (TZ), QT M2, and AR M2.

Specimen	Average Hardness	COF	Wear Volume (mm^3^)	Wear Rate (10^−5^ mm^3^/N·m)	Normalized Wear Resistance *
FSPed M2 (SZ)	896 ± 6	0.45 ± 0.02	0.15 ± 0.01	0.75 ± 0.05	14.0
FSP25% (TZ)	679 ± 78	0.50 ± 0.02	0.28 ± 0.04	1.40 ± 0.20	7.5
FSP50% (TZ)	699 ± 63	0.47 ± 0.03	0.24 ± 0.02	1.20 ± 0.10	8.8
FSP75% (TZ)	710 ± 32	0.50 ± 0.03	0.20 ± 0.01	1.00 ± 0.05	10.5
QT M2	779 ± 10	0.42 ± 0.03	0.12 ± 0.02	3.00 ± 0.50	3.5
AR M2	360 ± 3	0.55 ± 0.01	0.42 ± 0.06	10.50 ± 1.50	1.0

* Normalized wear resistance is defined as Wear rate of AR specimen/Wear rate of QT, single-pass and double-pass FSPed M2.

**Table 4:** E_corr_ and I_corr_ of FSPed M2, FSP25%, FSP50% and FSP75%, AR M2 and QT M2.

Specimens	E_corr_ (mV_SCE_)	I_corr_ (μA/cm^2^)
FSPed M2	−342.4 ± 2.7	4.5 ± 0.9
FSP25%	−397.6 ± 5.6	9.5 ± 0.8
FSP50%	−424.4 ± 6.0	14.7 ± 1.7
FSP75%	−440.9 ± 2.8	17.1 ± 1.9
QT M2	−430.7 ± 3.3	24.2 ± 1.5
AR M2	−441.5 ± 3.1	34.2 ± 1.0

**Table 5:** The fitting electrochemical parameters (EIS) of FSPed M2, FSP25%, FSP50% and FSP75%, AR M2 and QT M2.

Specimen	R_s_ (Ω·cm^2^)	CPE		R_ct_ (Ω·cm^2^)	χ^2^
		α (0–1)	Q_0_/(Ω^−1^ cm^−2^ s^α^)		
AR M2	5.85	0.795	0.0061038	898.70	0.000222
QT M2	7.84	0.685	0.0010131	1629.56	0.00125
FSPed M2	5.45	0.735	0.00039844	3647.03	0.00123
FSP75%	9.85	0.835	0.00055001	937.85	0.0104
FSP50%	7.96	0.813	0.00041941	1805.98	0.0401
FSP25%	10.89	0.797	0.00039977	1957.55	0.0541

## Data Availability

The original contributions presented in the study are included in the article. Further inquiries can be directed to the corresponding author.

## Abbreviations

The following abbreviations are used in this manuscript:

FSP	Friction stir processing
FSW	Friction stir welding
HSS	High-speed steel
HCP	Hexagonal close packing
UTS	Ultimate tensile strength
EDS	Energy disperse spectroscopy
FCC	Face center cubic
BCT	Body-centered tetragonal
BCC	Body-centered cubic
OZ	Overlapped zone
TZ	Tempered zone
SZ	Stir zone
T1	First track
T2	Second track
TMAZ	Thermal mechanically affected zone
HAZ	Heat affected zone
BM	Base material
AR M2	As-received AISI M2 HSS
QT M2	Quenched and tempered M2
FSP25%	Multiple-pass FSPed M2 with overlapping ratio of 25%
FSP50%	Multiple-pass FSPed M2 with overlapping ratio of 50%
FSP75%	Multiple-pass FSPed M2 with overlapping ratio of 75%
NETD	Noise equivalent temperature difference
XRD	X-ray diffraction
TWI	The Welding Institute
HEA	High entropy alloy
OR	Overlapping ratio
SEM	Scanning electron microscope
TEM	Transmission electron microscope
RS	Retreating side
AS	Advancing side
COF	Coefficient of friction
SCE	Saturated calomel electrode
OCP	Open circuit potential
PD	Potentiodynamic polarization
EIS	Electrochemical impedance spectroscopy
Ecorr	Corrosion potential
Icorr	Corrosion current
CPE	Constant phase element
CR	Corrosion resistance
